# Clinical Utility of Protein Induced by Vitamin K Absence-II in Patients with Hepatocellular Carcinoma

**DOI:** 10.31557/APJCP.2021.22.6.1731

**Published:** 2021-06

**Authors:** Abu Bakar H Bhatti, Kiran Naz, Ghazanfar Abbas, Nusrat Y Khan, Haseeb H Zia, Imran N Ahmed

**Affiliations:** 1 *Department of Hepato-Pancreatic-biliary Surgery and Liver Transplantation, Shifa International Hospital, Islamabad, Pakistan. *; 2 *Shifa Tameer-e-Millat University, Islamabad, Pakistan. *; 3 *Department of Pathology, Shifa International Hospital*,* Islamabad Pakistan. *

**Keywords:** PIVKA II- AFP, hepatocellular carcinoma, liver transplant, microvascular invasion

## Abstract

**Objective:**

Despite moderate sensitivity, alpha fetoprotein (AFP) is widely used in screening and prognostication for hepatocellular carcinoma (HCC). The objective of the current study was to assess clinical utility of Prothrombin induced by Vitamin K absence-II (PIVKAII) in addition to AFP in patients with HCC.

**Methods:**

We retrospectively reviewed 244 patients with documented AFP, PIVKA II and dynamic imaging of the liver. Using ROC curves, cutoff values for AFP and PIVKAII for HCC detection, tumor grade and microvascular invasion (MVI) were assessed. In patients who underwent liver transplantation (LT) for HCC, survival was determined using Kaplan Meier curves.

**Results:**

The median PIVKAII in healthy living donors was 28.6mAU/ml (15.9-55). In cirrhotics, the sensitivity of an AFP cutoff of 7.6 ng/ml or PIVKAII cutoff of 250 mAU/ml for HCC detection was 91.7% (176/192) and specificity was 62.9%(68/108) (P<0.0001). In patients with HCC, PIVKAII values were significantly elevated with tumor size > 5 cm (P < 0.0001), tumor nodules > 3(P=0.01), and macrovascular invasion(P<0.0001). The high risk group (patients with AFP ≥ 40 ng/ml + PIVKAII ≥ 350 mAU/ml), had a sensitivity of (23/33) 69.6% and specificity of (22/22)100% for MVI (P <0.001). The estimated 3 year RFS after LT in the low risk group (AFP <40 ng/ml or PIVKAII< 350 mAU/ml) was 100% versus 63%(P=0.001). The estimated 3 year RFS based with and without MVI was 80% and 100% (P=0.03).

**Conclusion:**

PIVKAII in combination with AFP, appears to have a promising role in surveillance and prognostication for HCC. It also allows improved patient selection for liver transplantation.

## Introduction

Hepatocellular carcinoma (HCC) is the 6th most common cancer worldwide and the 4th most common cause of cancer related mortality (Globocan, 2018). At presentation, a small percentage of patients are eligible for curative treatment. This is due to advanced tumor stage, degree of liver failure and poor performance status of patients (Bhatti et al., 2016).

Liver resection and transplantation are the two most effective treatment modalities in patients with HCC (Forner et al., 2013). Historically, tumor related factors such as tumor size and number, and severity of liver failure have been used to make treatment decisions in patients with HCC. While alpha-fetoprotein (AFP) has well established role in surveillance and prognostication of HCC, it has its own limitations (Hsu et al., 2018). For example, the sensitivity of AFP is only 40-60% for HCC detection [Heinbach et al., 2018; Gao and Song., 2017). Another biomarker, Protein induced by vitamin K absence-II (PIVKAII) appears to have a promising role in detection and prognosis of HCC. It has been included in the screening protocols of various guidelines(Ertle et al., 2013; Chinese Society of clinical oncology., 2018). However, there is inconclusive data on what should be considered a normal PIVKAII in healthy individuals, and PIVKAII cutoffs for surveillance and prognostication in patients with HCC remain unclear (Kudo et al., 2011; Yu et al., 2017). As far as curative treatment for HCC is concerned, presence of microvascular invasion (MVI) remains one of the most critical factors for treatment failure. There are no reliable markers to detect MVI preoperatively and the role of PIVKAII in predicting MVI also remains to be exclusively studied [Kim et al., 2016 ; Wu et al., 2018). In our center, from the year 2017 onwards, PIVKAII levels were assessed routinely along with AFP in patients with cirrhosis. In addition, PIVKAII testing was randomly performed in healthy voluntary liver donors to determine normal distribution in our population. 

The objective of the current study was to determine clinical utility with ofPIVKAII in addition to AFP in cirrhotic patients with HCC. 

## Materials and Methods

This was a review of patients seen at the department of Hepato-pancreatico-biliary surgery and liver transplantation, between April 2017 and June 2019. Patients with liver cirrhosis and documented pre treatment AFP and PIVKAII levels were included (n=244). Patients who did not have underlying cirrhosis were excluded (n=4). Presence of liver cirrhosis and HCC was confirmed on a dynamic CT scan of the liver or MRI. In doubtful cases, elastography was performed to confirm underlying fibrosis. All patients were discussed in multi disciplinary meeting and a treatment plan was formalized. To assess PIVKAII values in healthy individuals with no underlying liver disease, PIVKAII testing was performed in 60 potential liver donors who underwent donor workup in the year 2017. All potential donors underwent extensive workup which has been detailed elsewhere (Dar et al., 2016). Donors were healthy individuals, between 18-50 years of age, BMI <32 kg/m^2^, and had no underlying liver disease. The quantitative analysis of both AFP and PIVKAII was performed on electrochemiluminescence assay using COBAS 8000e602 analyzers. Previously, the normal reported PIVKAII values in Japanese and US population are < 40 mAU/ml and 63 mAU/ml respectively (Kasahara et al.,1993; Marrero et al.,2009). 

For the purpose of this study, we looked at median PIVKAII values in healthy donors and assessed the impact of various donor characteristics on normal PIVKAII levels. We also assessed AFP and PVKAII levels in patients with underlying cirrhosis. In addition, we looked at distribution of demographics, body mass index (BMI), etiology, model for end stage liver disease (MELD) scores, and Child Turcot Pugh (CTP) scores in cirrhotic patients. Receiver operator curves (ROC) were used to determine AFP and PIVKAII cutoffs for HCC in patients with underlying cirrhosis. For HCC detection, we opted for ROC cutoffs with high sensitivity (Kim et al., 2016). For the diagnosis of HCC, a liver dynamic CT scan or MRI was performed, demonstrating characteristic imaging findings of HCC (Willat et al., 2017). In patients with HCC, we assessed AFP and PIVKAII cutoffs for aggressive tumor features including tumor size > 5 cm, tumor nodules > 3, macrovascular invasion, and extra hepatic metastasis. Poor grade and microvascular invasion(MVI) are well known prognostic markers in patients with HCC (Kim et al., 2016). In our cohort, 50 patients underwent living donor liver transplantation (LDLT) for HCC. In this subgroup of patients, ROC analysis was performed to look for AFP and PIVKAII cutoffs for MVI and poor grade. For MVI or poor grade, we opted for ROC cutoffs with high specificity. It has been shown previously, that in patients with high risk HCC features, ROC curves with high specificity allow more patients to be eligible for curative treatments (Kim et al., 2016). Survival analysis was performed using Kaplan Meier curves in patients who underwent LDLT. Recurrence free survival (RFS) was calculated from date of transplant to the date of documented recurrence. Patients who died within index hospital admission were excluded from analysis for RFS(n=4). 

For categorical data, Chi square and Fischer test were used while for interval data t test or Mann Whitney U test was used. A P value < 0.05 was considered statistically significant. All analysis was performed on Statistical package for social sciences (SPSS) version 20. The study was approved by the ethics committee of Shifa International Hospital Islamabad (IRB # 221-1041-2020). 

## Results

*Healthy individuals*

Mean age was 27.88 ± 7.6 years in healthy donors (n=60). Male to female ratio was 4.9:1(203/41). The median PIVKAII value was 28.6(15.9-55)mAU/ml. There were 10 donors with PIVKAII values > 40mAU/ml (upper limit of normal reported in Japan) but all had values within range reported from United States. There was no significant difference in PIVKAII levels based on gender i.e. 28.8 and 25.6 mAU/m(P=0.58). On ROC analysis, donor age (P=0.5), BMI (P=0.2), and total bilirubin (P=0.9) were not significant for PIVKAII level > 40 mAU/ml.

*HCC negative versus HCC positive cirrhotics*

Mean age was 46.9 ± 11.5 and 54.6 ± 10 years (P <0.001) in HCC positive (n=176) and HCC negative (n=68) cirrhotics. There were significant differences between the HCC positive and the HCC negative patients with regards to age < 50 i.e. 62 (35.2%) vs 36 (52.9%)(P=0.01), HCV etiology 139 (79%) vs 29 (42.6%) (P< 0.0001), CTP class C 26 (14.8%) vs 31(45.5%) ((P<0.001) and ≤ 14 MELD score 116 (65.9%) vs 31 (45.5%) (P<0.001) as shown in Table 1. 

**Figure 1:**
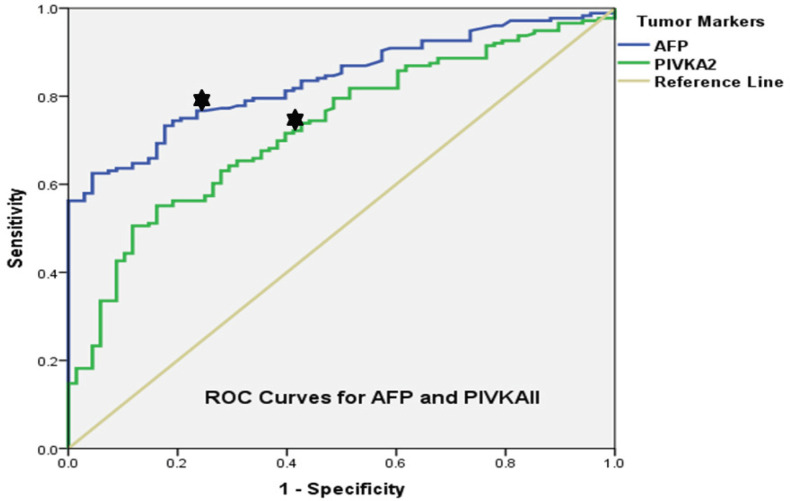
ROC Curves for AFP and PIVKAII Level Cutoffs for HCC Detection in Cirrhotic Patients. PIVKAII cutoff = 250 mAU/ml (sensitivity 72%, specificity 60% (AUC=0.72, P<0.001). AFP cutoff= 7.6 ng/ml (sensitivity 77%, specificity 77% (AUC=0.83, P<0.001)

Median PIVKAII level was 1194.2(5.6-30000) mAU/ml and155.4(18.4-13681.3)(P<0.001) in HCC positive and negative cirrhotics. Median AFP level was 45.6 (0.7-114229) ng/ml and 4.7(1.5-29.6)(P<0.001) respectively. Using ROC curves, a PIVKAII cutoff of 250 mAU/ml had 72% sensitivity and 60% specificity (AUC=0.72, P<0.001) for HCC as shown in Figure 1. An AFP cutoff of 7.6 ng/ml had a sensitivity and specificity of 77% (AUC=0.83, P<0.001). When combined, an AFP cutoff of 7.6 ng/ml and PIVKAII cutoff of 250 mAU/ml had a sensitivity of 91.7% (176/192) and specificity of 62.9%(68/108)(P<0.001) for HCC in cirrhotics. The positive predictive value (PPV) was (176/176+40) 81.4% and the negative predictive value (NPV) was (68/68+16) 80.9%. 

*Poor prognostic factors in HCC*

Table 2 demonstrates median AFP and PIVKAII levels for various tumor related characteristics. 

A significant difference was seen for tumor size > 5 cm (P < 0.001), tumor number > 3(P=0.01), and macrovascular invasion(P<0.001). No significant difference was noted in median AFP and PIVKAII values in patients with extra hepatic metastasis.

*Tumor markers and microvascular invasion*

**Figure 2:**
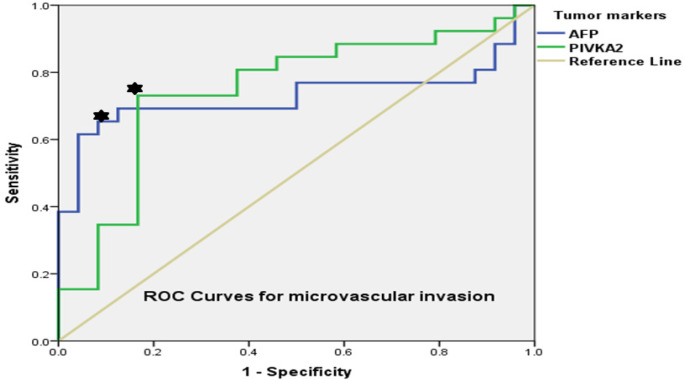
ROC Curves for AFP and PIVKAII for Detection of Microvascular Invasion. PIVKAII cutoff = 350 mAU/ml (sensitivity=73%, specificity 83% (AUC=0.74, P=0.003); AFP cutoff = 40 ng/ml (sensitivity=66%, specificity= 88% (AUC=0.72, P=0.005)

On ROC analysis, AFP level and PIVKAII level showed no significance for well-moderate versus poor tumor grade (AUC=0.6, P= 0.2) and (AUC= 0.51, P= 0.8). However, a PIVKAII cutoff of 350 mAU/ml had 73% sensitivity and 83% specificity (AUC=0.74, P=0.003) while an AFP cutoff of 40 ng/ml had 66% sensitivity and 88% specificity (AUC=0.72, P=0.005) for MVI as shown in Figure 2.

**Figure 3:**
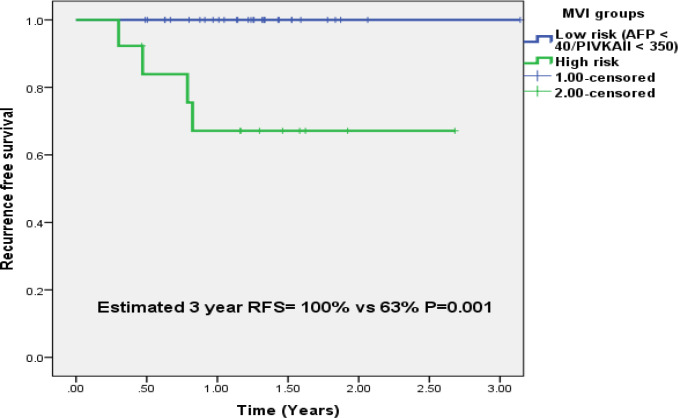
Estimated 3 Year Recurrence Free Survival in Patients with Low and High Risk Groups Based on AFP and PIVKAII Levels

The high risk group (patients with AFP ≥ 40 ng/ml + PIVKAII ≥ 350 mAU/ml), had a sensitivity of (23/33) 69.6% and specificity of (22/22)100% for MVI (P <0.001). The PPV was (23/23+0)100% and negative predictive value was (22/22+10)68.5%. During the follow up period, no recurrence was seen in the low risk group (n=33) while 4/13(30.7%) patients had a recurrence in the high risk group. The estimated 3 year RFS in the high risk group was 63% versus 100% in the low risk group (P=0.001) as shown in Figure 3. 

**Figure 4:**
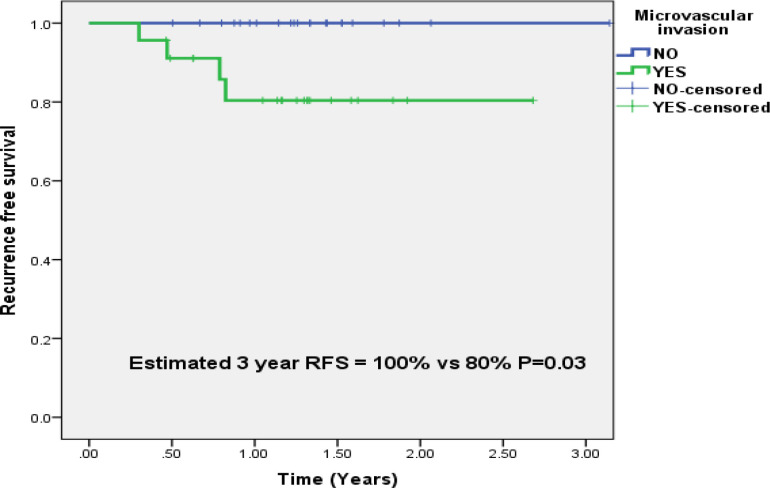
Estimated 3 Year Recurrence Free Survival in Patients with and without Microvascular Invasion

In patients who underwent LDLT for HCC (n=45), there was no recurrence in patients who were negative for MVI on explant histopathology(n=22), while 4/23(17.3%) patients with MVI had recurrence. The estimated 3 year RFS based on MVI was 100% versus 80% (P=0.03)(Figure 4). 

**Table 1:** Patient Characteristics in Cirrhotic Patients with and without Hepatocellular Carcinoma

		Cirrhosis HCC present (n=176) N (%)	Cirrhosis HCC absent (n=68) N(%)	P value
Gender	Male	150(85.2)	53(77.9)	0.1
Age group (years)	<50	62(35.2)	36(52.9)	0.01
Body mass index (Kg/m^2^)	<25	75(42.6)	27(39.7)	0.3
Etiology	HCV	139(79)	29(42.6)	<0.001
	HBV	23(13)	16(23.5)	
	HCV+HBV	13(7.4)	12(17.6)	
	Others	1(0.6)	11(16.1)	
CTP class	A	87(49.4)	16(23.5)	<0.001
	B	63(35.8)	21(30.9)	
	C	26(14.8)	31(45.5)	
MELD score	≤ 14	116(65.9)	31(45.5)	0.004

**Table 2:** Median AFP and PIVKAII Values for Tumor Related Factors in Patients with Hepatocellular Carcinoma

		Median AFP (range)	P value	Median PIVKAII (range)	P value
Tumor size (cm) (n=115)	< 5	27.9 (0.7-20000)	0.002	410 (15.8-30000)	<0.001
(n=61)	>5	192.1 (2.1-114229)		3196 (5.6-30000)	
Tumor number(n=117)	< 3	30.3 (0.7-114229)	0.01	684 (16-30000)	0.01
(n=59)	> 3	90.9 (2.3-20000)		2804 (5.6-30000)	
Macrovascular invasion (n=119)	No	25.3 (0.7-30000)	0.004	455 (5.6-30000)	<0.001
(n=57)	Yes	158.1 (1.4-114229)		4632 (25-30000)	
Extra hepatic metastasis (n=156)	No	44.1 (0.7-114229)	0.8	918 (5.6-30000)	0.2
(n=20)	Yes	102.3 (2.76-20000)		1194 (5.6-30000)	

## Discussion

This is the first study to assess PIVKAII levels in healthy Pakistani population. The levels in healthy voluntary liver donors were higher than previously reported from Japan but comparable to the US population. Among cirrhotic patients, the combined use of AFP and PIVKAII, for detection of HCC had high sensitivity and good PPV. Our results showed consistency with other reports and AFP and PIVKAII were both significantly elevated in patients with larger tumors, more tumor nodules and macrovascular invasion. We found AFP and PIVKAII to be useful in predicting microvascular invasion in surgical candidates. 

Serum bio markers such as AFP and PIVKAII are considered to have low sensitivity for detecting HCC. Both biomarkers are useful in advanced stages of HCC with aggressive features. As a result, there is controversy regarding most effective screening protocols for HCC in high risk patients. Different societies recommend varying combinations of imaging and biomarkers for HCC detection (Burak et al., 2015; Balaceanu et al., 2019). While ultrasound (US) has certain limitations including operator dependency and low sensitivity in cirrhotics, AFP and PIVKAII when combined, might offer high sensitivity rates in these patients.

Liver resection and transplantation remain the most effective treatments for patients with HCC and underlying cirrhosis. While presence of liver failure and portal hypertension limits wide spread utility of hepatic resections, liver transplantation cures both HCC and liver failure. MVI is one of the most important predictors of post transplant recurrence in patients with HCC (Rodríguez-Perálvarez et al., 2013). Our inability to reliably detect MVI preoperatively, with imaging or biopsy, compromises ideal patient selection for transplantation (Park et al., 2017). Consequentially, tumor related factors such as tumor size and number have been used to select appropriate candidates for treatment. Despite being restrictive, Milan criteria remains the bench mark for liver transplantation in HCC (Mazzaferro et al., 1996). However, it has been shown that with careful selection, comparable outcomes can be achieved in patients outside Milan criteria (Bhatti et al., 2019).One such approach involves increasing reliability on bio markers to select optimal candidates for liver transplantation(Kim et al., 2016;Duvoux et al., 2012).

In LDLT, more liberal cutoffs on tumor size, number and AFP are used. In such settings, the ability to rule out MVI can greatly help in improving post transplant outcomes. In this regard, role of pre transplant imaging in detecting MVI remains limited (Reginelli et al., 2018).The association between MVI and biomarkers like PIVKAII has been previously explored, predominantly in the setting of hepatic resections and merits further validation in LDLT(Shirabe et al., 2007; Kaibori et al., 2010; Yamashita et al., 2018; Amado et al., 2019; Shindoh et al., 2014).As shown in our patient cohort, none of the MVI negative patients developed recurrence in the follow up period. We found a combined AFP of 40 ng/ml and PIVKAII of 350 mAU/ml to be 100% specific for microvascular invasion with 100% PPV. Moreover, it allowed improved segregation of patients into low and high risk groups irrespective of tumor size or number. With low risk of MVI, LDLT can be potentially offered to patients with otherwise more aggressive tumor related features. 

There are certain limitations of the current study. We used dynamic imaging of the liver as the gold standard for the diagnosis of HCC. It is possible that some patients were under or over staged due to inherent limitations in imaging. Nevertheless, dynamic imaging is the standard worldwide for HCC detection. Our cohort included patients who were seen in the hospital, mostly due to complications of cirrhosis. This might represent a slightly different patient group when compared with screen detected patients in the community. Nevertheless, The combined use of AFP and PIVKAII was associated with high sensitivity and merits further exploration for screening high risk patients. 

In conclusion, when combined with AFP, PIVKAII appears to be a useful biomarker for HCC detection in high risk patients. Higher values are associated with aggressive tumor features like larger tumor size, number of tumor nodules and macrovascular invasion. Notably, PIVKAII has a promising role in excluding MVI in HCC patients and might be a good tool for selecting patients for surgical therapies. In the future, biomarkers will have expanding role in selecting treatment options for patients with HCC. Role of PIVKAII needs to be further explored in larger studies and different patient cohorts .

## Author Contribution Statement

AHB contributed to design, methodology, manuscript writing and critical review KN, GA contributed to data collection and manuscript writing NYK, HHZ and INA contributed to design and critical review of the manuscript.
